# The NOGO receptor NgR2, a novel αVβ3 integrin effector, induces neuroendocrine differentiation in prostate cancer

**DOI:** 10.1038/s41598-022-21711-5

**Published:** 2022-11-07

**Authors:** Fabio Quaglia, Shiv Ram Krishn, Khalid Sossey-Alaoui, Priyanka Shailendra Rana, Elzbieta Pluskota, Pyung Hun Park, Christopher D. Shields, Stephen Lin, Peter McCue, Andrew V. Kossenkov, Yanqing Wang, David W. Goodrich, Sheng-Yu Ku, Himisha Beltran, William K. Kelly, Eva Corey, Maja Klose, Christine Bandtlow, Qin Liu, Dario C. Altieri, Edward F. Plow, Lucia R. Languino

**Affiliations:** 1 Prostate Cancer Discovery and Development Program, Thomas Jefferson University, Philadelphia, PA USA; 2 Department of Pharmacology, Physiology, and Cancer Biology, Thomas Jefferson University, Philadelphia, PA USA; 3 Department of Medicine, School of Medicine, MetroHealth Medical Center, Rammelkamp Center for Research, Case Western Reserve University, Cleveland, OH USA; 4 Cardiovascular and Metabolic Sciences Department, Lerner Research Institute, Cleveland Clinic, Cleveland, OH USA; 5 Department of Pathology, Thomas Jefferson University, Philadelphia, PA USA; 6 Center for Systems and Computational Biology, Wistar Institute, Philadelphia, PA USA; 7 Department of Pharmacology and Therapeutics, Roswell Park Comprehensive Cancer Center, Buffalo, NY USA; 8 Department of Medical Oncology, Dana-Farber Cancer Institute and Harvard Medical School, Boston, MA USA; 9 Department of Medical Oncology, Thomas Jefferson University, Philadelphia, PA USA; 10 Department of Urology, University of Washington, Seattle, WA USA; 11 Institute of Neurochemistry, Biocenter, Medical University of Innsbruck, Innsbruck, Austria; 12 Molecular and Cellular Oncogenesis Program, The Wistar Institute, Philadelphia, PA USA; 13 Immunology, Microenvironment and Metastasis Program, The Wistar Institute, Philadelphia, PA USA

**Keywords:** Prostate cancer, Oncology

## Abstract

Androgen deprivation therapies aimed to target prostate cancer (PrCa) are only partially successful given the occurrence of neuroendocrine PrCa (NEPrCa), a highly aggressive and highly metastatic form of PrCa, for which there is no effective therapeutic approach. Our group has demonstrated that while absent in prostate adenocarcinoma, the αVβ3 integrin expression is increased during PrCa progression toward NEPrCa. Here, we show a novel pathway activated by αVβ3 that promotes NE differentiation (NED). This novel pathway requires the expression of a GPI-linked surface molecule, NgR2, also known as Nogo-66 receptor homolog 1. We show here that NgR2 is upregulated by αVβ3, to which it associates; we also show that it promotes NED and anchorage-independent growth, as well as a motile phenotype of PrCa cells. Given our observations that high levels of αVβ3 and, as shown here, of NgR2 are detected in human and mouse NEPrCa, our findings appear to be highly relevant to this aggressive and metastatic subtype of PrCa. This study is novel because NgR2 role has only minimally been investigated in cancer and has instead predominantly been analyzed in neurons. These data thus pave new avenues toward a comprehensive mechanistic understanding of integrin-directed signaling during PrCa progression toward a NE phenotype.

## Introduction

Integrins are transmembrane receptors composed of two non-covalently linked subunits (α and β) and, as indicated by various studies, show altered distribution during prostate cancer (PrCa) progression^1–3^. The αVβ3 integrin is usually present at low levels in normal human prostate cells but is highly expressed in advanced PrCa; it promotes invasion and adhesion of cancer cells to extracellular matrix proteins, such as vitronectin^3,4^. The activation state of the αVβ3 integrin, the affinity/avidity for cognate ligands, is regulated by Kindlin-2 (K2, Mig-2, *FERMT2*)^5–7^, which interacts with the cytoplasmic tail of the β3 subunit^8–10^. K2 is expressed in many different cell types and plays an essential role during integrin-dependent interaction between cells and the extracellular matrix^11^.

Recently, we have reported that the αVβ3 integrin is highly expressed in human and mouse neuroendocrine prostate cancer (NEPrCa) but absent in prostate adenocarcinoma (ADPrCa)^12,13^. In contrast, another αV integrin, αVβ6, is present in ADPrCa^14^ but is negligible in NEPrCa^13^. NEPrCa is a highly aggressive and metastatic subtype of PrCa that typically develops from subsets of castrate-resistant PrCa (CRPrCa) cells^15^. NEPrCa develops either de novo or through the acquisition of alterations in pre-existing epithelial tumors in response to androgen deprivation therapies^15–17^. The NE phenotype appears to result from cells that do not express androgen receptor (AR) or prostate-specific antigen (PSA) but instead express neuron-specific proteins, such as synaptophysin (SYP), neuron-specific enolase (NSE), and chromogranin A (CHGA)^18,19^. These aberrations activate pro-tumorigenic pathways independently from those downstream of the AR^20,21^. A recent study suggests that other changes, in addition to AR signaling loss, are necessary for neuroendocrine differentiation (NED) to take place^22^. The role of the αVβ3 integrin in NEPrCa has not been investigated.

The Nogo receptor family is formed by three structurally related molecules: NgR1, NgR2, and NgR3. NgR2 core protein (45 kDa), in this study its sialylated form (48 kDa), or glycosylated forms (65 kDa) are predominantly detected^23^. NgRs are glycosylphosphatidylinositol (GPI)-anchored receptors that lack both the transmembrane and the intracellular domains^24,25^. This protein family is characterized by eight leucine-rich repeats (LRR) flanked by the N- and C-terminal cysteine-rich regions. The C-terminal LRR domain is connected to the GPI-anchor for the membrane attachment via a stalk region^24,25^. Despite its name, NgR2 does not bind to Nogo^26^, but it is known to bind to myelin-associated glycoprotein (MAG)^23,25^. Members of the NgR family form a signal transduction complex with the nerve growth factor receptor p75^24,27^ and LINGO-1^28^ that activates RhoA^29^. RhoA is a member of the Ras superfamily of small GTPases that, in cancer, regulates cytoskeletal dynamics to mediate cell migration^30^. In PrCa, elevated RhoA levels have been associated with aggressive disease and decreased disease-free survival after radical prostatectomy^31^. In addition, enzalutamide-resistant PrCa cells express higher levels of RhoA compared to their enzalutamide-sensitive counterparts^32^. Finally, activation of RhoA by the neuropeptide bombesin stimulates PC3 cell migration^33^.

Here we demonstrate that the αVβ3 integrin, known to be upregulated in NEPrCa^13^, increases the levels of a GPI-anchored receptor called NgR2 (Nogo-66 receptor homolog 1) in NEPrCa cells. The role of the NgR protein family, to the best of our knowledge, has been minimally investigated in cancer^34–36^, as it has been predominantly studied in neurons. Specifically, NgR2 is reported in a correlative study to be associated with Hodgkin lymphoma^34^. We show here that NgR2 is significantly upregulated in NEPrCa patients’ tumors and NE cell lines and is co-expressed with NE markers in NEPrCa patient-derived xenografts (PDXs), and different NE mouse models. Moreover, from a mechanistic point of view, we show that NgR2 promotes NE differentiation, anchorage-independent growth, and cell motility. We also show that the αVβ3 integrin immunoprecipitates with NgR2. Finally, the αVβ3 integrin has to be activated by K2 in order to induce NgR2 upregulation. Our results show that NgR2 is a novel effector of the αVβ3 integrin that promotes NED in PrCa and contributes to the highly motile phenotype of NEPrCa.

## Materials and methods

### Cell lines

Culture conditions for the PrCa cell lines (C4-2B, LNCaP, PC3) have been previously described^4,37,38^. NCI-H660 cells were grown following ATCC instructions. PC3 cells were transfected using lentivirus shRNA clones to: Kindlin-2, clone TRCN0000128058 (which targets a coding sequence of K2); and non-targeting scrambled control SHC002 (purchased from Sigma). The lentivirus-mediated shRNA gene knockdown procedures were previously described in^39,40^. LNCaP cells were used for CRISPR/Cas9-mediated knockout of Kindlin-2 (*FERMT2*) as previously described^41^. Culture conditions for the PrCa cell lines 22Rv1 and VCaP were previously described^42^.

### Generation of Kindlin-2 Knockout cell lines using electroporation

The sgRNA pool of 3 guide RNAs (G*A*C*GGGAUAAGGAUGCCAGA, C*G*C*GGUUCAGGUCCGUCACA and A*G*G*CGUGAUGCUUAAGCUGG with their respective Synthego modified EZ scaffolds) targeting FERMT2 and the scramble control were obtained from Synthego. The sgRNA pellets were rehydrated in 1X TE buffer (provided by Synthego) to make a stock of 100 μM. A working solution of 30 μM sgRNA was made (in nuclease free water) fresh before electroporating the cells. For every reaction, the Ribonucleoprotein (RNP) complex was assembled by adding 3 μl of30 μM sgRNA to 0.5 μl of 20 μM Cas9 (provided by Synthego) at a ratio of 9:1 in 3.5 μl resuspension buffer R (Neon Transfection System; Invitrogen Basel, Switzerland) and incubated for 10 min at room temperature.

Electroporation of LNCaP cells was achieved by an implemented electroporation device system according to manufacturer’s instructions (Neon Transfection System; Invitrogen, Basel, Switzerland). The Neon Transfection System 10 μL kit was used for the transfection of human prostate cancer cells. Cells were cultured 48 h before electroporation and harvested at nearly 80% confluency. Cells at a density of 2 × 10^5^ were washed with PBS and resuspended in 5 μl resuspension buffer R (Neon Transfection System; Invitrogen Basel, Switzerland). Within 15 min of resuspension, the cells were added to the tube containing RNP and the cell-RNP complex was electroporated with the Neon Transfection System. Per electroporation, 2 × 10^5^ cells were taken up in a 10 μl Neon tip using the Neon Transfection System pipette (Invitrogen). The electroporation was performed by applying 3 pulses at 1450 Volts for 10 ms to PC3 cells and 2 pulses at 1200 Volts for 20 ms to LNCaP cells. Control cells were incubated with the resuspension buffer without the sgRNA and electroporated at the same settings. After electroporation, the cells were seeded in a 6-well plate by adding 2.5 ml DMEM (Cytiva) with 10% FBS without antibiotic supplements. The cells were cultured for 48–72 h and subsequently proceeded for further analysis. Western blot analysis was used to assess the Kindlin-2 KO efficiency in all cell lines.

### Antibodies

The following antibodies (Abs) were used: for the immunoblotting (IB) analysis, rabbit monoclonal Abs against the αVβ3 integrin (13166S, Cell Signaling) and Aurora Kinase A (14475S, Cell Signaling), polyclonal goat Abs against the αVβ6 integrin (AF2389, R&D system) and NgR2 (AF2776, R&D system), rabbit polyclonal Abs against calnexin (CANX, sc11397, Santa Cruz), actin (a2066, Sigma), RhoA (sc-179, Santa Cruz), TSG101 (Abcam, ab30871), mouse monoclonal Abs against RhoA (sc-418, Santa Cruz), NSE (LS-C197136, LSBio), Kindlin-2 (MAB2617, Millipore), were also used. For immunohistochemical analysis, rabbit monoclonal Ab against the β3 integrin (13166S, Cell Signaling), rabbit polyclonal Abs against SYP (PA1-1043, Invitrogen), and NgR2 (PA5-98577, Invitrogen) were also used. Rabbit IgG (I5006, Sigma) was used as negative control. For immunoprecipitation, rabbit polyclonal Ab against NgR2 (PA5-98577, Invitrogen), rabbit monoclonal Ab against the β3 integrin (13166S, Cell Signaling), mouse monoclonal Abs against the β6 (62A1) and β1 integrins (NBP2-52708, Novus) were used. For the adhesion assay, the αVβ3 integrin (LM609, Millipore MAB1976), and the non-immune mouse IgG (02-6502, Thermo Fisher) were used.

### Immunoprecipitation

PC3 cells were lysed with lysis buffer (50 mM Tris–HCl pH 7.2, 150 mM NaCl, 1% Triton X-100, 1 mM Na3VO4, 1 mM Na4O7P2, 50 mM NaF, 0.01% aprotinin, 4 μg/ml pepstatin, 10 μg/ml leupeptin, 1 mM PMSF, 1 mM CaCl_2_, 1 mM MgCl_2_, 1 μM Calpain inhibitor) and pre-clearing was performed by two consecutive incubations with protein G-Sepharose (17061801, Cytivia) at 4 °C for 30 min. Binding to specific Abs was performed by incubation at 4 °C overnight, followed by incubation with protein G-Sepharose at 4 °C for 3 h. After six washes with lysis buffer, immunocomplexes were resuspended in 1X reducing Laemmli buffer and separated by SDS-PAGE. All western blotting films were developed using the Protec Optimax developer system. The film’s images were acquired using a Microtek ArtixScan M2 and processed using the Microtek Scan Wizard Pro V8.20 software.

### PrCa cell transfection

PC3 cells were transfected with three different shRNA constructs that target *RTN4RL2* (SMARTvector, Dharmacon/Horizon, SO-2914049G, sequences: V3SVHS00_4716901 for sh*RTN4RL2*_1, and V3SVHS00_8801245 for sh*RTN4RL2*_3). As a control, PC3 cells were transfected with a non-targeting scrambled control shRNA (SMARTvector, Dharmacon/Horizon, VSC11707). DU145 cells were transfected with pCMV6-Entry vector carrying RTN4RL2 (Origene, SC310413) or with the empty vector as a control (Origene, PS100001). Cells were plated (2.5 × 10^5^) in a 6-well plate and grown overnight at 37 °C. The next day, cells were washed with PBS and incubated with 1 mL serum-free media at 37 °C for 2 h. For transfection, 4 μg of plasmid was mixed with 12 μL of lipofectamine 2000 (Invitrogen, 11668-019) in 200 μL of serum-free RPMI media. The plasmid-lipofectamine mix was incubated at room temperature for 25 min. The mix was then added drop-wise to the cells and incubated at 37 °C for 6 h. After 6 h, 700 μL of growth medium (without pen-strep) was added to the cells and incubated at 37 °C overnight. After 24 h, the medium was replaced with the growth medium, and cells were incubated for 48 h at 37 °C. For the selection of the transfected cells, the growth medium was supplemented with puromycin (3 μg/mL) for PC3 cells and G418 (0.5 mg/ml) for DU145 cells. The pooled populations of selected cells were maintained in complete media containing 2 μg/mL of puromycin for PC3 cells and 0.5 mg/ml of G418 for DU145 cells.

### siRNA transfection

**Figure 1:**
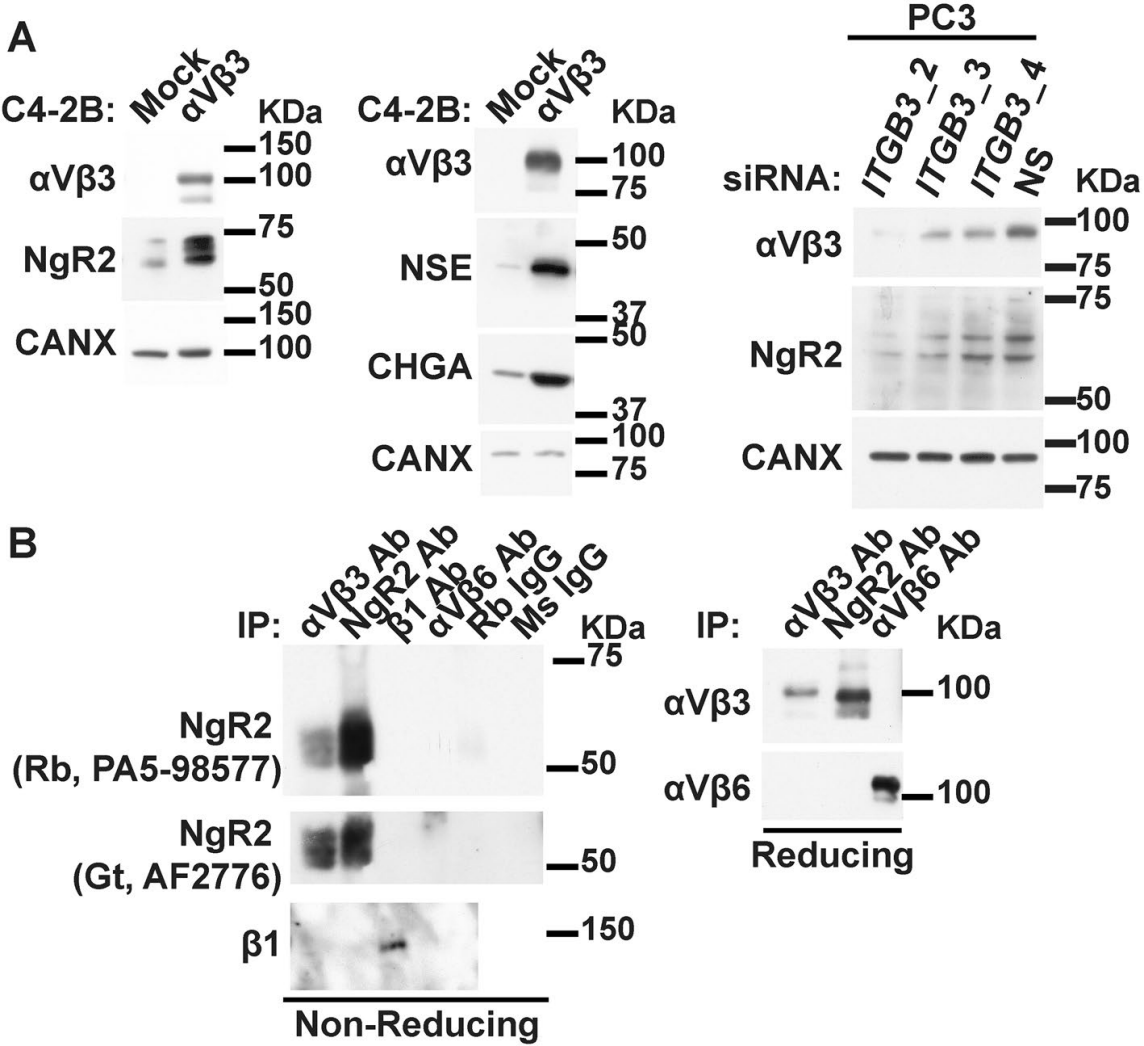
The αVβ3 integrin increases NgR2 expression and is associated with NgR2. (**A**) Immunoblotting analysis of the expression levels of the αVβ3 integrin and NgR2 (n = 3, left panels), and of the αVβ3 integrin, the NE markers chromogranin A (CHGA) and neuron-specific enolase (NSE; n = 1, middle panels), in C4-2B cells that exogenously express αVβ3 or their mock control cells. Right panel, immunoblotting analysis of the expression levels of the αVβ3 integrin, and NgR2 in PC3 cells in which the αVβ3 integrin expression was downregulated using three different siRNAs against *ITGB3* (the gene responsible for the β3 integrin subunit). Non-silencing siRNA (NS) was used as control. Calnexin was used as loading control. (**B**) PC3 cell lysates were immunoprecipitated with Abs against the αVβ3 integrin, NgR2, the β1 integrin, the αVβ6 integrin or their isotype controls (left panel) and the αVβ3 integrin, NgR2, and the αVβ6 integrin (right panel). Immunoprecipitates were analyzed for NgR2, the β1 integrin, the αVβ3 integrin, and the αVβ6 integrin by immunoblotting (n = 2). Immunoblotting analyses were performed under non-reducing (left panel) and reducing conditions (right panel). NgR2 was detected using two different Abs (left panel).

Downregulation of *RTN4RL2* (NgR2) expression was accomplished using siRNA SMARTPool (L-008045-00-0010, Dharmacon/Horizon). For the downregulation of *ITGB3* FlexiTube (Qiagen) siRNAs were used. In Fig. 1: siRNA_2, SI00004599 (target sequence, CTCTCCTGATGTAGCACTTAA), siRNA_3, SI00004606 (target sequence, CAAGCTGAACCTAATAGCCAT), and siRNA_4, SI02623159 (target sequence, CACGTGTGGCCTGTTCTTCTA) were used.

Transfection of the siRNA and immunoblotting analysis were performed as previously described^14^. Briefly, 300,000 cells were transfected with siRNA (final concentration 100 nM) duplexes using oligofectamine at a final concentration of 20 nM. Twenty-four hours after transfection, the siRNAs were removed, and the cells were kept in complete media overnight. This process was repeated for a second time, after which the cells were harvested and analyzed.

### RNA sequencing

The heatmap of gene expression in cell lines was generated using Broad Institute Morpheus software (MA, USA). Statistical analysis was done using GraphPad Prism (CA, USA) and differences between two groups were compared by unpaired student's t-test.

### RNA sequencing of metastatic CRPrCa samples

RNA sequencing analysis of metastatic CRPrCa specimens acquired through rapid autopsy of 98 patients was performed as previously described^43^. Specimens were classified based on their levels of AR and NE markers. AR-positive and NE-negative (n = 76), AR low NE-negative (n = 13), AR-positive NE-positive (n = 9), AR-negative NE-negative (n = 8), and AR-negative NE-positive (n = 11).

### Generation of mice carrying prostate-specific deletions

Mice of genotype PB-Cre4 *pten*^*loxP/loxP*^* rb1*^*loxP/loxP*^* trp53*^*loxP/loxP*^ (TKO), and PB-Cre4 *pten*^*loxP/loxP*^ (SKO) were generated as previously described^44^. Mice with the same genetic background (mixed C57BL/6 and 129SVJ) were used for the wild-type control.

### Generation of NgR2 knockout mice

NgR2-null, and NgR1-null mice have been previously described^45–47^. Wild-type (WT) littermate controls, NgR2^−/−^ and NgR1^−/−^ animals were obtained by mating heterozygous NgR2^+/−^ and NgR1^+/−^ animals, respectively.

### Animal care

Care of animals followed standards established by the Office of Laboratory Animal Welfare, NIH, Department of Health and Human Services. All mice were maintained following the recommendations of the Institutional Animal Care and Use Committee which is a standing committee mandated by Federal law and regulations that ensures the humane and ethical treatment of animals. All experimental protocols were approved by the Animal Care and Use Committees at the institutions where the mice were hosted: Thomas Jefferson University for CB-17 SCID mice; Roswell Park Cancer Center for TKO mice and the Medical University of Innsbruck, Austria for NgR2-null and NgR1-null mice. This study is reported in accordance with ARRIVE guidelines.

### Immunohistochemical analysis

Immunohistochemical analysis was performed on tissue sections of 5 TKO (primary tumor and lung metastases), and LuCaP PDXs TMA as previously described^13^. The tissue sections were incubated overnight at 4 °C with Abs against the β3 integrin subunit (1:25), NgR2 (1:500 for mouse samples and 1:1000 for LuCaP PDXs TMA), SYP (1:200), or the IgG isotype, which was used as the negative control. The following day, the tissue sections were washed with PBST (5 min × 2), followed by PBS (5 min), and incubated with biotinylated goat anti-rabbit IgG (BA-1000, Vector Laboratories) in PBST for 30 min at room temperature. Pictures were acquired using an Olympus BX43 microscope and the imaging processing was performed using the Olympus Sens Entry V2.3 software.

The specificity of our IHC staining was confirmed by staining dorsal root ganglion sections samples from three NgR2 knockout mice (Fig. S1A^45^) or by preincubating for 1 h the NgR2 Ab (Invitrogen, PA5-98577) with the blocking peptide (DSRGRQGGDAPTEDDYWG, 10 μg/ml, Thermo Fisher) that is specifically recognized by this Ab (Fig. S1B). The preincubated Ab was then used for immunostaining of rat brain samples or primary tumors from a NEPrCa patient (Fig. S1B).

### Human subject inclusion criteria

The NEPrCa tissue sample was obtained from the Department of Pathology at Thomas Jefferson University (Philadelphia, PA). The specimen was de-identified and discarded in accordance with guidelines established by the Institutional Review Board (IRB), an administrative body established to protect the rights and welfare of human research subjects recruited to participate in research activities conducted at Jefferson.

### LuCaP TMA immunohistochemical assessment and statistical analysis

The immunostaining of each LuCaP was scored as previously reported^13^.

### MTT assay

PC3 cells treated with oligofectamine, non-silencing siRNA, or siRNA specifically targeting *RTN4RL2* were plated (10^4^) in 100 µL complete media on 96 well plates (six replicates) and analyzed using 3-(4,5-dimethylthiazol-2-yl)-2,5-diphenyltetrazolium bromide (MTT; 5 mg/mL).

### Boyden chamber assay

PC3 cells treated with oligofectamine, non-silencing siRNA, or siRNA specifically targeting *RTN4RL2* were seeded (5 × 10^4^) on fibronectin-coated (10 µg/ml) Transwell chambers (three replicates) in serum-free media and analyzed as been previously described^38^.

### Anchorage-independent growth assay

Six-well plates were coated with 0.8% agarose to create a bottom layer and 10,000 PC3 cells (parental, scramble, or sh*RTN4RL2*) from each well were resuspended in 2 mL of complete medium (RPMI containing 10% FBS, 100 μg/mL streptomycin, and 100 U/mL penicillin). The protocol has been previously described^12^. Pictures were acquired using a Nikon Eclipse TS100 microscope and the imaging processing was performed using the NIS Elements F3.0 software.

### Adhesion assay

Adhesion assays were performed as previously described^48^.

### αVβ3 activation assay

Activation assays were performed as previously described^49^.

## Results

### The αVβ3 integrin increases NgR2 levels and is associated with NgR2 in PrCa cells

Our previous studies have shown that αVβ3 expression correlates with NEPrCa phenotype^13^. Therefore, we investigated mechanisms that would be selectively altered by αVβ3 expression. We find that the exogenous expression of the αVβ3 integrin induces a significant increase of NgR2 levels, as well as the NE markers chromogranin A (CHGA) and neuron-specific enolase (NSE) in C4-2B and LNCaP cells (Fig. 1A, left and middle panels, and Fig. S2). Moreover, when the expression levels of the αVβ3 integrin are reduced using siRNA against *ITGB3* (the gene responsible for the αVβ3 integrin) the NgR2 protein levels are reduced as well (Fig. 1, right panel) demonstrating that NgR2 expression is under the control of the αVβ3 integrin. These data suggest that NgR2 is a downstream effector of the αVβ3 integrin that may promote PrCa progression toward a NE phenotype.

In order to investigate if NgR2 interacts with the αVβ3 integrin, we performed immunoprecipitation of NgR2, and the αVβ3, αVβ6, and β1 integrins in PC3 cells. The immunoblotting analysis shows that NgR2 co-precipitates in a unique manner with the αVβ3 integrin but not with the αVβ6 integrin nor with the β1 integrins (Fig. 1B). We then conclude that NgR2 may act as a cofactor that would affect αVβ3 function by associating with this integrin.

### The αVβ3/NgR2 pathway is upregulated in human and mouse NEPrCa

**Figure 2:**
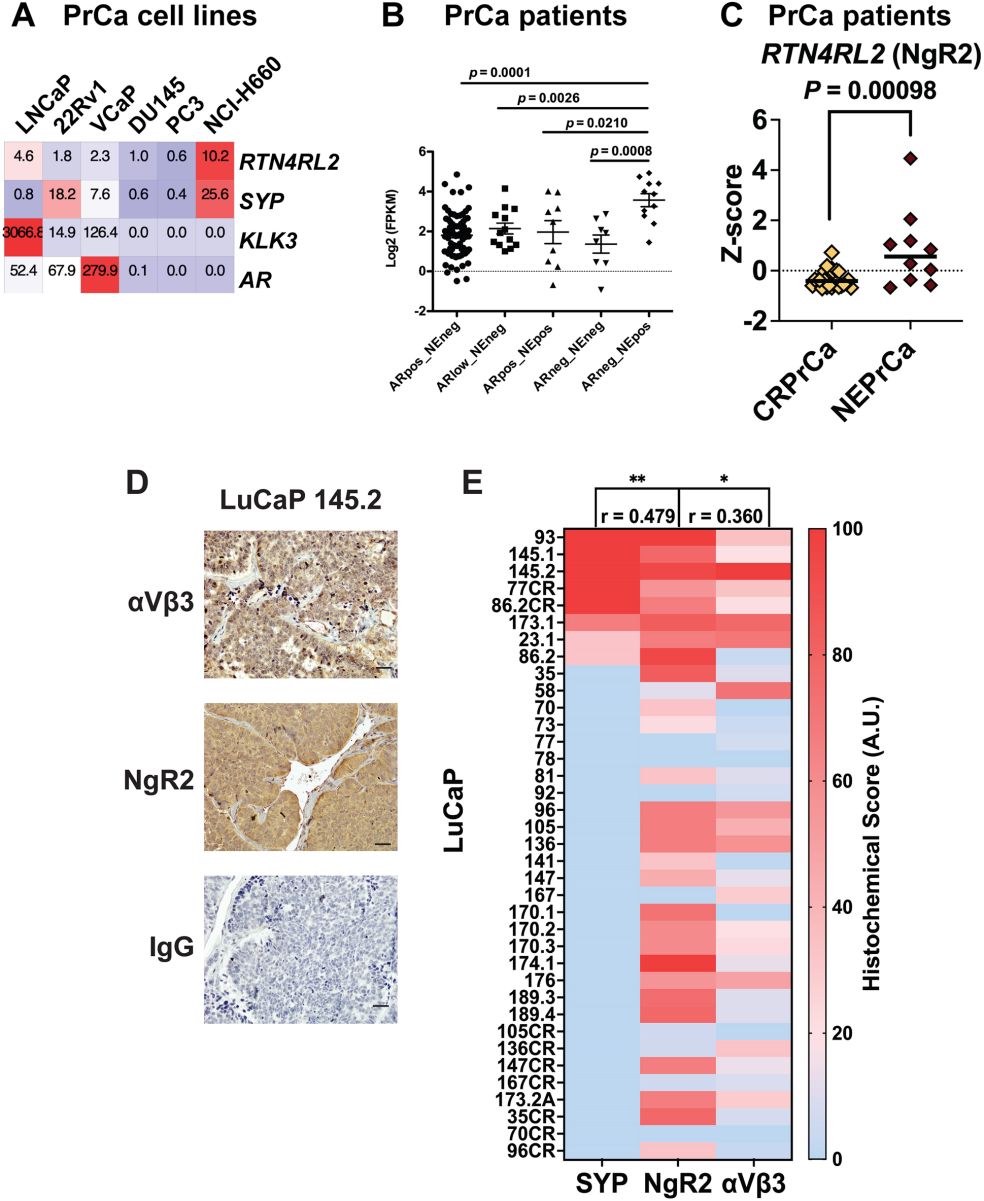
NgR2 expression is increased in NEPrCa patients, NE cell lines, and NEPrCa PDXs. (**A**) Left panel, mRNA levels for *RTN4RL2*, *SYP* (Synaptophysin), *KLK3* (Kallikrein Related Peptidase 3), and *AR* (Androgen Receptor) reported as Transcripts Per Million were compared across multiple PrCa cell lines in the CCLE database^50^. (**B**) RNA sequencing analysis for *RTN4RL2* of metastatic CRPrCa specimens acquired through rapid autopsy of 98 patients. Specimens are classified based on their levels of AR and NE markers. AR-positive NE-negative (ARpos_NEneg, n = 76), AR low NE-negative (ARlow_NEneg, n = 13), AR-positive NE-positive (ARpos_Nepos, n = 9), AR-negative NE-negative (ARneg-NEneg, n = 8), and AR-negative NE-positive (ARneg_NEprs, n = 11). Statistical analysis was performed using GraphPad Prism (CA, USA) and differences between two groups were compared using unpaired student’s t-test. (**C**) RNA sequencing analysis of *RTN4RL2* expression for CRPrCa and NEPrCa tumors from cBioPortal (dataset Neuroendocrine Prostate Cancer, Multi-Institute^20^). Differential expression between the two groups is estimated by student’s t-test. (**D**) Representative IHC staining for the αVβ3 integrin or NgR2 of LuCaP PDX TMAs (n =37) is shown. IgG was used as negative control. The bar at the bottom right corner of each panel represents 20 μm. (**E**) Heat map of the histochemical score for SYP, NgR2, and the αVβ3 integrin of each LuCaP is shown. *P = 0.029; **P = 0.003. Spearman correlation was performed and r-values are reported. The histochemical score for the αVβ3 integrin and SYP has been previously reported^13^.

To determine if NgR2 expression is relevant in NEPrCa we compared different prostate cancer cell lines in the Cancer Cell Line Encyclopedia (CCLE) database^50^. NCI-H660, a NEPrCa cell line, expresses high *RTN4RL2* (the gene encoding for NgR2) mRNA levels compared to the AR positive cell lines LNCaP, 22Rv1, and VCaP as well as the AR negative cell lines, DU145 and PC3 (Fig. 2A). The 22Rv1 cell line instead maintains *AR* as well as *SYP* expression but low levels of *RTN4RL2* (NgR2). The gene for *SYP* (synaptophysin) was used as a NE marker, and the genes for *AR* and *KLK3* (Kallikrein Related Peptidase 3) were used as markers of the AR activity. Screening of the University of Washington metastasis dataset^43^ and classification of the metastatic CRPrCa samples by their expression levels of AR and NE markers shows increased levels of *RTN4RL2* (NgR2) in those samples that have similar characteristics as NEPrCa tumors (ARneg_NEpos) compared to the AR-positive NE-negative samples, AR low NE-negative samples, AR-positive NE-positive samples, and AR-negative NE-negative samples (Fig. 2B). We then analyzed RNA-seq data for NEPrCa and CRPrCa tumors from cBioportal (dataset Neuroendocrine Prostate Cancer, Multi-Institute^20^) and estimated whether a differential expression between the two groups is observed. The t-test results show a significantly higher expression of *RTN4RL2* (NgR2) in NEPrCa samples compared to the CRPrCa samples (Fig. 2C). In contrast, the other two members of the NgR family (NgR1 and NgR3) do not show differential expression between NEPrCa and CRPrCa that passed correction for multiple testing (FDR < 10% threshold; Fig. S3). In particular, *RTN4RL1* (NgR3) does not exhibit significant differences between the two groups (*P* = 0.12), while *RTN4R* (NgR1), although it has significance with a *P*-value of 0.008, shows FDR = 12%, compared to NgR2 *P* = 0.00098, FDR = 6%. These results suggest a unique role of NgR2 in the NgR family during cancer progression and metastasis in patients with NEPrCa.

To further support the involvement of NgR2 in NEPrCa, we assessed the presence of NgR2 using immunohistochemical analysis (Fig. 2D) and scored the immunostaining intensity and percentage of cells at each staining level of 37 LuCaP PDXs^43,51^ in the tumor microarrays (TMAs) using the scoring system described in the Materials and Methods section and previously reported^13^. These PDX models were generated by implanting primary PrCa or metastatic lesion tumor fragments from PrCa patients into immunocompromised mice^51^, and the resulting PDX models were subsequently characterized for their expression of NE markers^43^. We previously reported a positive correlation between the αVβ3 integrin and the NE marker, SYP (*r* = 0.420; *P* = 0.0046)^13^ in these LuCaP PDXs. Our immunohistochemical analysis now shows a positive correlation between NgR2 and the αVβ3 integrin (*r* = 0.360, *P* = 0.029), as well as between NgR2 and the NE marker, SYP (*r* = 0.479, *P* = 0.003) (Fig. 2E).

**Figure 3:**
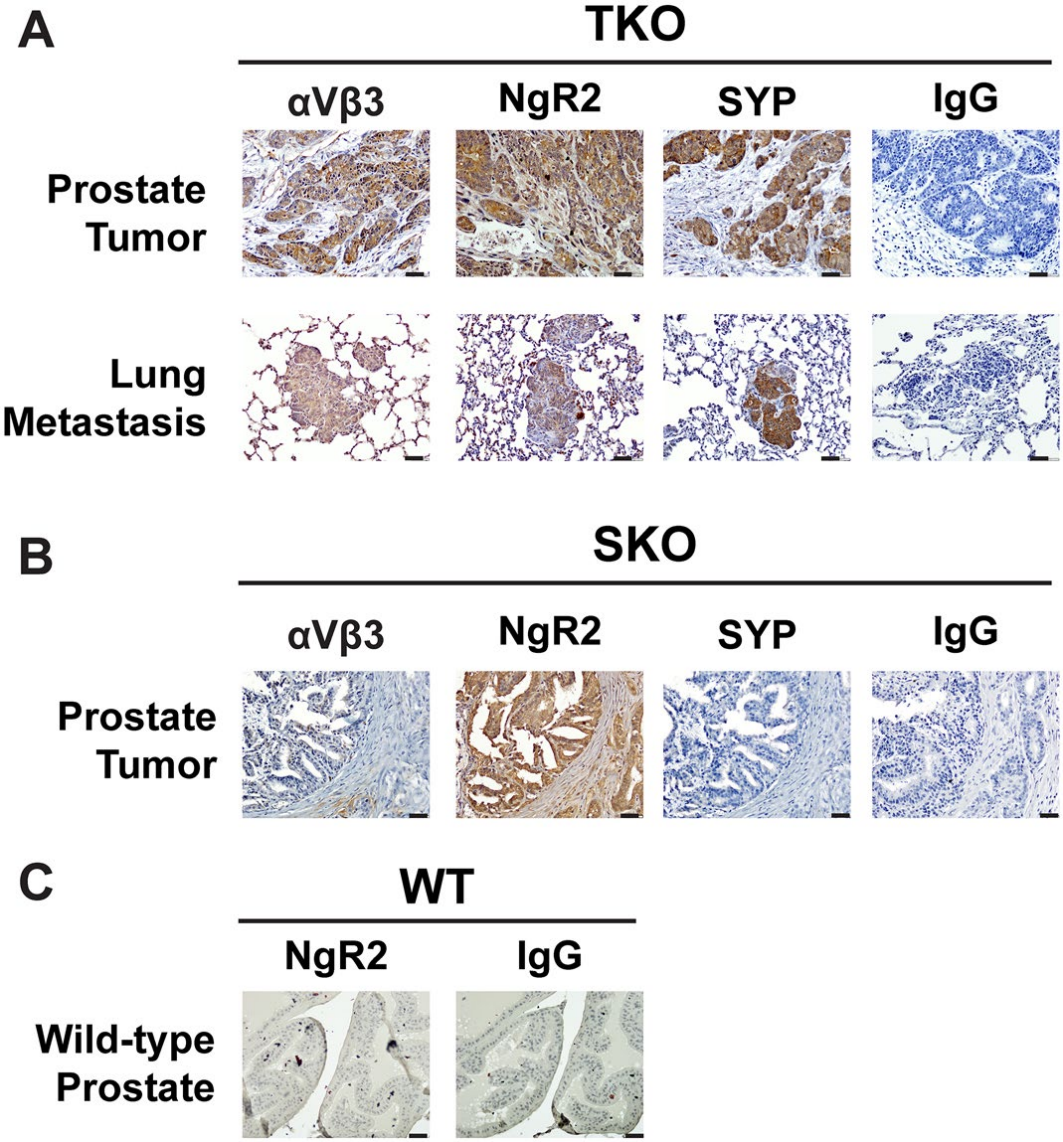
NgR2 expression is increased in NEPrCa TKO mouse tumors. (**A, B**) Representative immunohistochemical staining of the αVβ3 integrin, NgR2, and SYP of prostate tumors (top row) and lung metastasis (bottom row) from TKO mice (**A**, n = 5) and of primary tumors from SKO mice (**B**, n = 5). (**C**) Representative immunohistochemical staining of NgR2 of prostate samples from wild-type mice (n = 4). IgG was used as negative control (**A–C**). The bars at the bottom right corner of each panel represent 20 μm.

In addition, we assessed the levels of NgR2 and the αVβ3 integrin in primary tumors and metastatic lung lesions from NEPrCa mice carrying *pten, rb1,* and *trp53* triple conditional deletions in the prostatic epithelium (PBCre4 *pten*^*loxP/loxP*^* rb1*^*loxP/loxP*^*trp53*^*loxP/loxP*^*,* TKO). This model develops NEPrCa similarly to its human counterpart^44^. The immunostaining analysis reveals co-expression of NgR2 and the αVβ3 integrin (Fig. 3A) in SYP-positive TKO tumors. Moreover, we analyzed tumors from a single conditional knockout mouse model (PBCre4 *pten*^*loxP/loxP*^, SKO) that develops adenocarcinoma tumors of the prostate. NgR2 is still detectable in these tumors (Fig. 3B). These tumors do not express αVβ3 or NE markers, indicating the requirement of both αVβ3 and NgR2 for NED. Finally, we show that NgR2 is undetectable in prostate samples from wild-type mice, known to be αVβ3 negative^13^ (Fig. 3C).

These results confirm the presence of NgR2 in human NE tumors and metastases, NEPrCa PDXs, the NEPrCa cell line NCI-H660, and NE tumors of TKO mice, further supporting our hypothesis that NgR2 has a role during PrCa progression toward a NE phenotype and metastasis.

### NgR2 controls NE marker expression and anchorage-independent growth in PrCa cells

**Figure 4:**
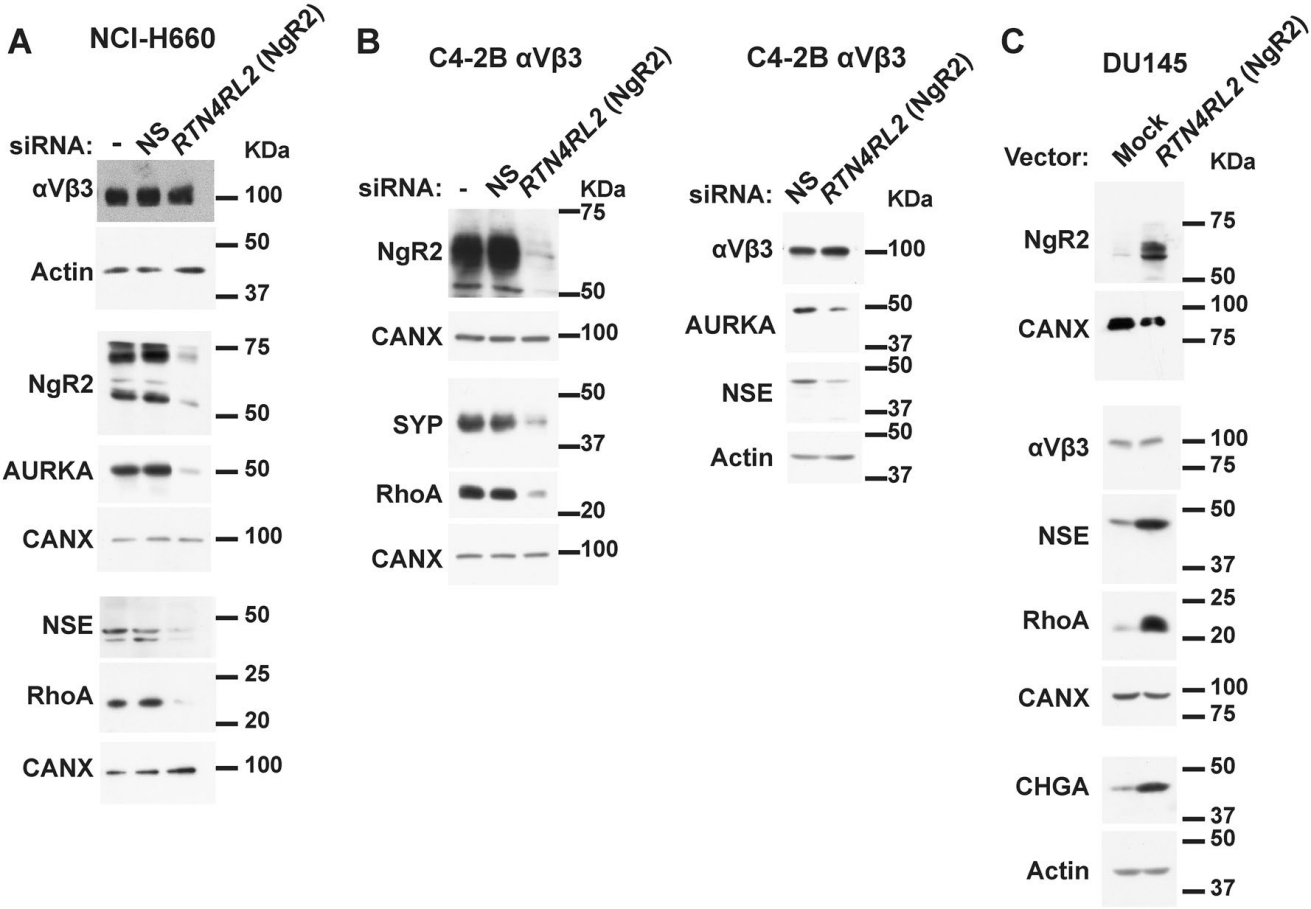
NgR2 induces NE markers and RhoA in CRPrCa and NEPrCa cells in vitro. (**A**) Immunoblotting analysis of the expression levels of the αVβ3 integrin (on a 7.5% PAGE), NgR2 and Aurora Kinase A (AURKA, on a 7.5% PAGE), and neuron-specific enolase (NSE) and RhoA on a 12.5% PAGE in NCI-H660 cells in which NgR2 expression was downregulated using a pool of siRNAs against *RTN4RL2* (NgR2). AURKA and NSE protein expression, measured using densitometric analysis, is reduced 11.22 and 8.4 fold, respectively, when compared to the non-silencing siRNA control (n = 4). (**B**) Left panel, immunoblotting analysis of the expression levels of NgR2 (on a 7.5% PAGE); RhoA and synaptophysin (SYP, on a 10% PAGE) in C4-2B cells that exogenously express αVβ3 in which NgR2 expression was downregulated using a pool of siRNA against *RTN4RL2. *Right panel, immunoblotting analysis of the expression levels of the αVβ3 integrin, AURKA, and NSE (on a 12.5% PAGE) in C4-2B cells that exogenously express the αVβ3 integrin and in which NgR2 expression was downregulated using a pool of siRNAs against *RTN4RL2* (n = 3). AURKA and NSE protein expression, measured using densitometric analysis, is reduced 3.59 and 3.9 fold, respectively, when compared to the non-silencing siRNA control. (**A,B)** –, Oligofectamine; NS, non-silencing. (**C**) Immunoblotting analysis of the expression levels of NgR2 (on a 10% PAGE), the αVβ3 integrin, NSE, and RhoA (on a 10% PAGE), and Chromogranin A (CHGA, on a 10% PAGE) in DU145 cells that exogenously express NgR2 or their mock control cells (n = 2). Actin or CANX was used as loading control (**A–C**). All immunoblotting analyses were performed under reducing conditions.

To further investigate the role of NgR2 during cancer progression and in cell motility, we reduced NgR2 expression in NCI-H660 cells (Fig. 4A) and C4-2B cells that exogenously express αVβ3 (Fig. 4B) using a pool of siRNAs (Fig. 4A, B) against *RTN4RL2* (NgR2). Upon NgR2 downregulation, our results show a reduced expression of the NE markers NSE, and SYP, as well as Aurora Kinase A (AURKA, Fig. 4A, B), which has been reported to be upregulated in NEPrCa^42,52^. RhoA, a known motility promoter^53^, and downstream effector of NgRs^54^ is also downregulated in both cell lines upon siRNA treatment (Fig. 4A, B left panel). On the other hand, when NgR2 is exogenously expressed in DU145 cells, the NE markers NSE and CHGA levels are upregulated (Fig. 4C). Moreover, RhoA is increased in these cells (Fig. 4C). Noticeably, the αVβ3 integrin is not increased by NgR2, suggesting that NgR2 is a downstream effector of this integrin. Taken together, these results suggest that NgR2 is involved in PrCa progression toward a NE phenotype.

**Figure 5:**
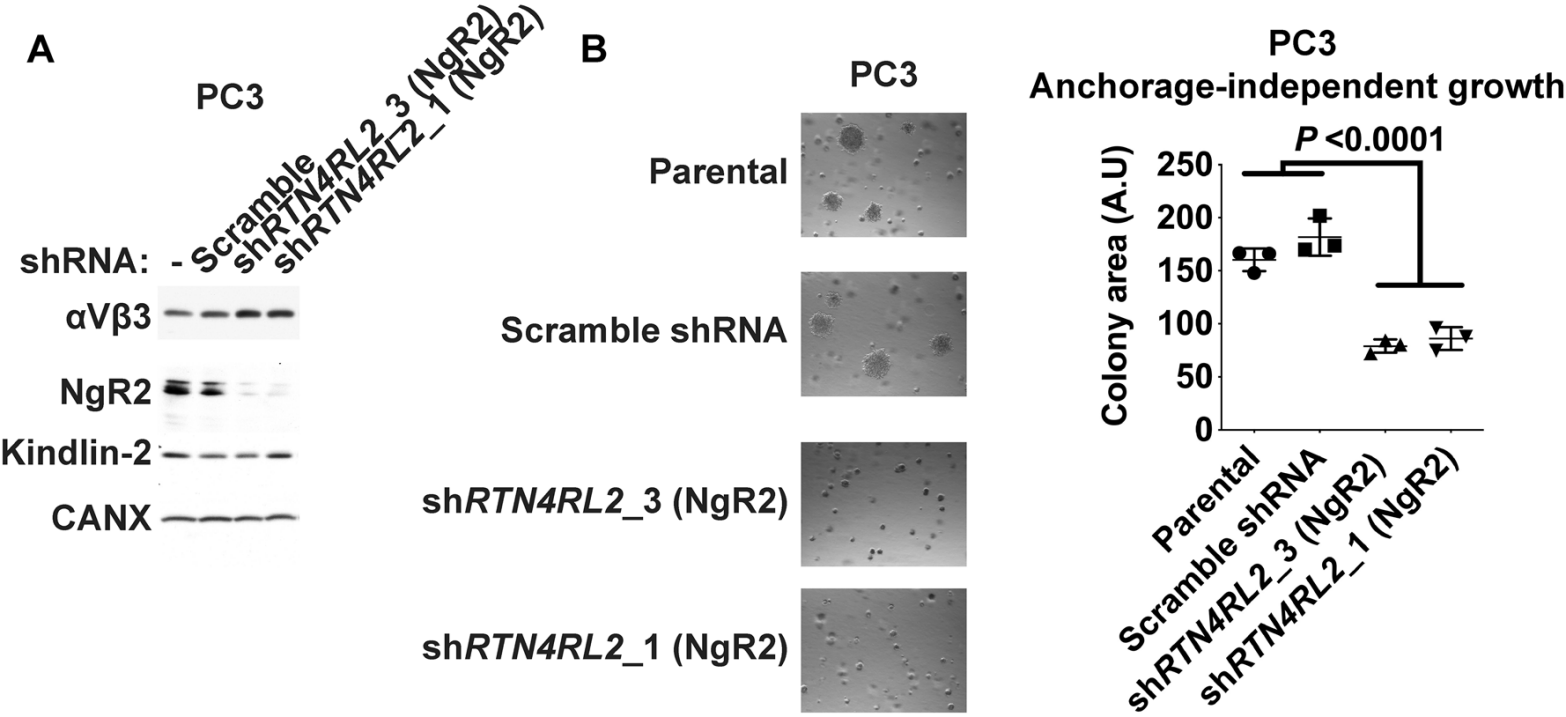
NgR2 increases the anchorage-independent growth of PC3 PrCa cells. (**A**) Immunoblotting analysis of the αVβ3 integrin, and NgR2 expression levels in different PC3 cell transfectants in which NgR2 is downregulated using shRNA against *RTN4RL2* (NgR2). CANX was used as loading control. The immunoblotting analysis was performed under reducing conditions. (**B**) Left panels, representative images of the colonies formed by the PC3 cells containing shRNA against *RTN4RL2* (NgR2), the scramble shRNA control, and the parental cells. Right panel, average colony area of the PC3 cells shown in (**A**) grown in 0.3% agar as described in the Materials and Methods section. Each condition has been replicated three times, and a total of nine fields per condition has been recorded. Values are reported as mean ± SEM. Significance was calculated by student’s t-test.

**Figure 6:**
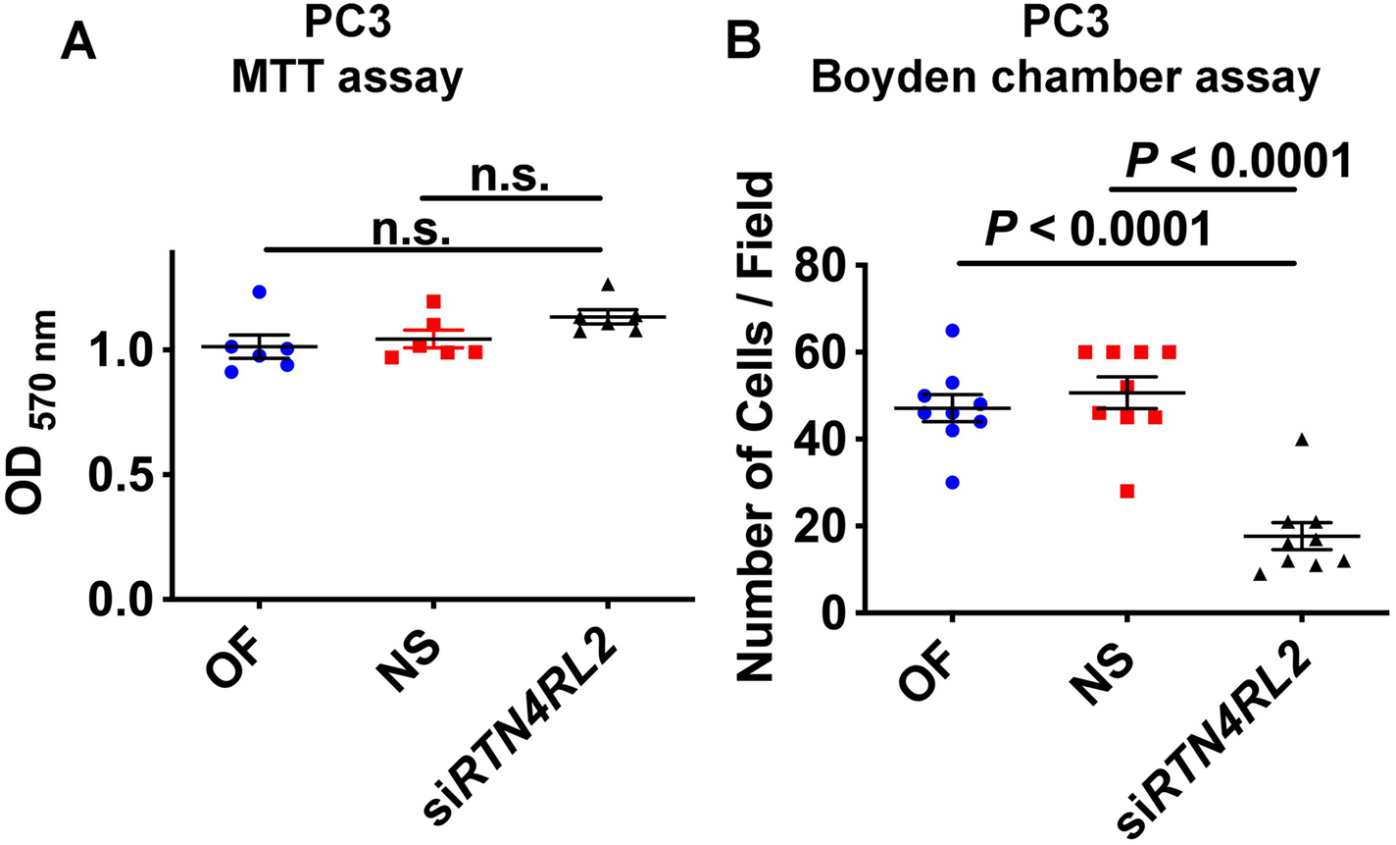
NgR2 modulates the motility but not the proliferation of PrCa cells. (**A**) PC3 cells, in which NgR2 expression was downregulated using a pool of siRNAs against *RTN4RL2* (NgR2), were plated on 96-well plates (1 × 10^4^, n = 6) in complete media. After 24 h, the viability of PC3 cells was determined by MTT assay; values are reported as mean ± SEM. (**B**) The PC3 cells were also plated (5 × 10^4^, n = 3) on fibronectin-coated (10 µg/ml) Transwell chambers in serum-free media in both top and bottom chambers as previously described^4^. The dot plot represents the number of PC3 cells migrated in each treatment group towards the bottom chambers in 6 h (9 fields for each condition, field of view = 0.055 mm diameter). Values are reported as mean ± SEM; *P*-values were calculated by unpaired t-test using GraphPad Prism. OF, Oligofectamine; NS, non-silencing; n.s., non-significant.

Next, we investigated the effects of NgR2 on anchorage-independent growth of PrCa cells. We stably knocked down NgR2 expression in PC3 cells using shRNA (Fig. 5A) and evaluated their anchorage-independent growth. As a control, we used scrambled shRNA transfected cells. In these cells, we do not detect significant differences in the expression levels of the αVβ3 integrin (Fig. 5A). The reduced expression of NgR2 results in a significant decrease of PC3 colony size (Fig. 5B), showing that NgR2 promotes anchorage-independent growth of PrCa cells. Moreover, we downregulated NgR2 using siRNA in PC3 cells and used these cells to analyze whether NgR2 affects cell proliferation and motility. As control, we used non-silencing RNA transfected cells. Our proliferation assay shows no significant differences upon reduced NgR2 expression (Fig. 6A). On the other hand, the motility of PC3 cells upon knockdown of NgR2 expression is significantly reduced (Fig. 6B). These results show that NgR2 promotes anchorage-independent growth and motility rather than the proliferation of PrCa cells, suggesting a potential role in metastasis formation.

Suppression of the αVβ3 integrin activation by Kindlin-2 downregulation reduces NgR2 expression levels.

**Figure 7:**
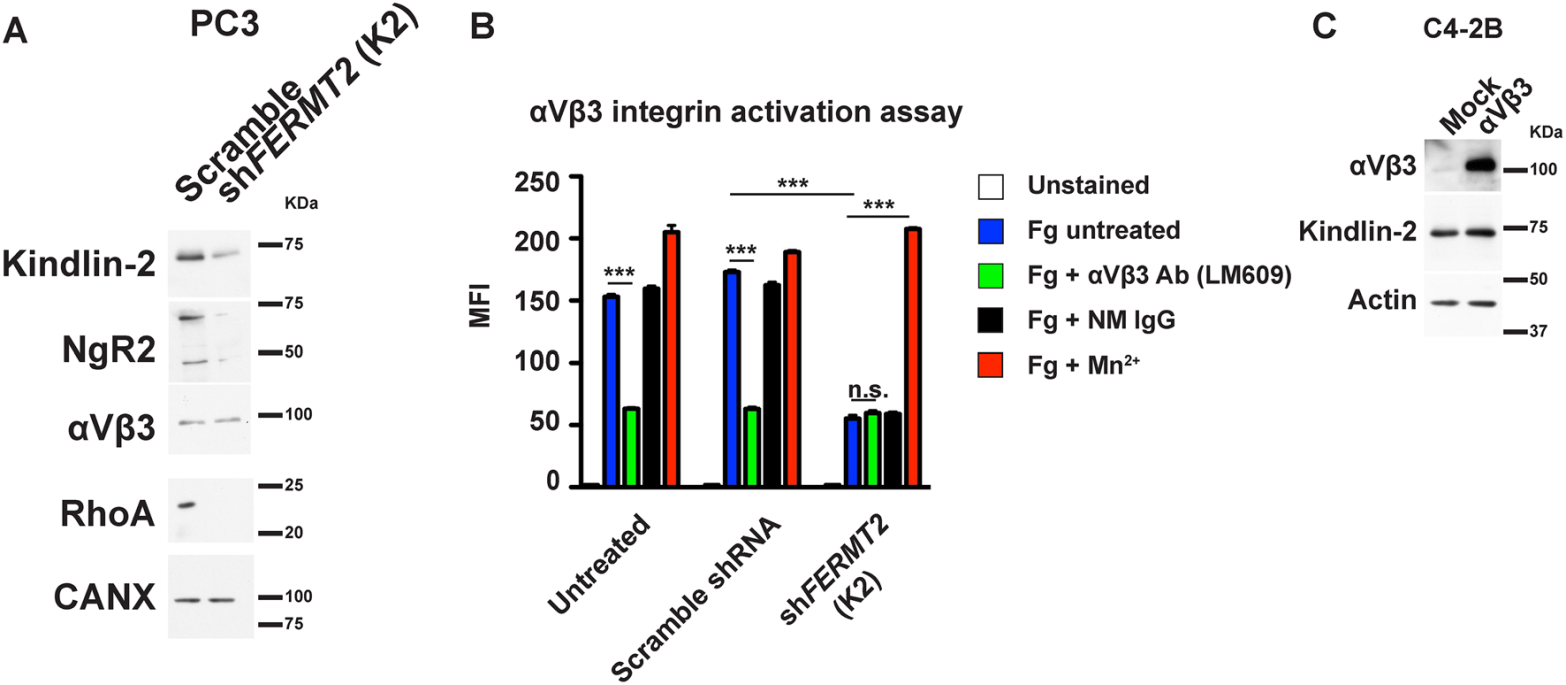
The αVβ3 integrin must be active to induce NgR2 in PrCa cells. (**A**) Immunoblotting analysis of the expression levels of Kindlin-2, NgR2, αVβ3, and RhoA in the lysates from PC3 cells in which Kindlin-2 expression was downregulated using shRNA targeting FERMT2 (the gene responsible for Kindlin-2). CANX was used as loading control. (**B**) Flow cytometry analysis of αVβ3 activation by its capacity to bind soluble Fg-Alexa Fluor 488 in PC3 cells transfected with a shRNA against FERMT2 or the non-targeting shRNA control (n = 2). Values are reported as Mean Fluorescence Intensity (MFI), and *P*-values were calculated by unpaired t-test using GraphPad Prism. (**C**) Immunoblotting analysis of the expression levels of the αVβ3 integrin and Kindlin-2 in C4-2B cells that express the αVβ3 integrin or their mock control. Actin was used as loading control. All immunoblotting analyses were performed under reducing conditions. n.s., non-significant; ***P < 0.001.

We then assessed whether increased expression of NgR2 requires the αVβ3 integrin to be in an activated state (high affinity/avidity for ligand). In order to de-activate the αVβ3 integrin, we downregulated the expression of the integrin co-activator K2 in PC3 and in LNCaP cells using shRNA or sgRNA, respectively (Fig. 7A and Fig. S4A). Our results show that NgR2 levels were reduced by K2 downregulation (Fig. 7A and Fig. S4A). We confirmed that when inactivation of the αVβ3 integrin activity is reduced by K2 downregulation, NgR2 levels are decreased as well. We confirmed the reduced activation of the αVβ3 integrin by assaying the ability of the PC3 cells to bind Fibrinogen (Fg) by flow cytometry and adhesion assay. When K2 expression levels were reduced by shRNA, the capacity of αVβ3 to specifically bind Fg was ablated (Fig. 7B and Fig. S4B). This binding is confirmed to be specific since Fg binding is inhibited by the function-blocking αVβ3 antibody LM609 (Fig. 7B and Fig. S4B). Our results confirm reduced PC3 cell binding in the absence of K2 due to the reduced αVβ3 integrin activity (Fig. 7B and Fig. S4B). In addition, we were able to bypass the inactivation of the αVβ3 integrin by incubating the K2 downregulated PC3 cells with Mn^2+^ (Fig. 7B) which has been reported to activate the αVβ3 integrin independently from K2 and inside-out signaling^55^.

## Discussion

In this study, we demonstrate that the αVβ3 integrin activates a signaling pathway in cancer cells that requires the expression of a GPI-linked surface molecule, NgR2, also known as Nogo-66 receptor homolog 1. We show here that NgR2 promotes NED, tumor growth, as well as a motile phenotype of PrCa cells; these findings are novel because NgR2 has minimally been investigated in cancer^34^ and has instead been predominantly analyzed in neuroscience studies.

Here we show that NgR2 levels are increased upon αVβ3 integrin expression and NgR2 co-precipitates with the αVβ3 integrin. To the best of our knowledge, this is the first time that an interaction between NgR2 and any integrin has been reported. We envision that an association between NgR2 and the αVβ3 integrin, shown in this study, may form a pro-tumorigenic and pro-metastatic complex in PrCa cells. Similar associations have been shown for members of the NgR family; when they bind to MAG, they form a signal transduction complex with the nerve growth factor receptor (NGFR) p75^24,27^. This receptor is known to drive phenotype switching in melanoma^56^ and has been recently demonstrated to promote melanoma metastasis when released in extracellular vesicles^57^. Similarly, NgR2 might stimulate an aggressive phenotype in PrCa through p75 in a complex with the αVβ3 integrin. We thus speculate that neuronal vesicular NgR2 may also contribute to cancer cell invasion; by co-opting a mechanism used by nerves, which promotes neural invasion of cancer cells in a paracrine manner^58^, NgR2 may be taken up by cancer cells, associate with the αVβ3 integrin and promote PrCa progression. Finally, cellular context may affect NgR2 association with the αVβ3 integrin since previous studies have shown that NgR2 binding to either MAG or to a proteoglycan, versican, inhibits the functions of adult neurons^46,59^ or stimulates activities in the neurons of newborns^60,61^.

**Figure 8:**
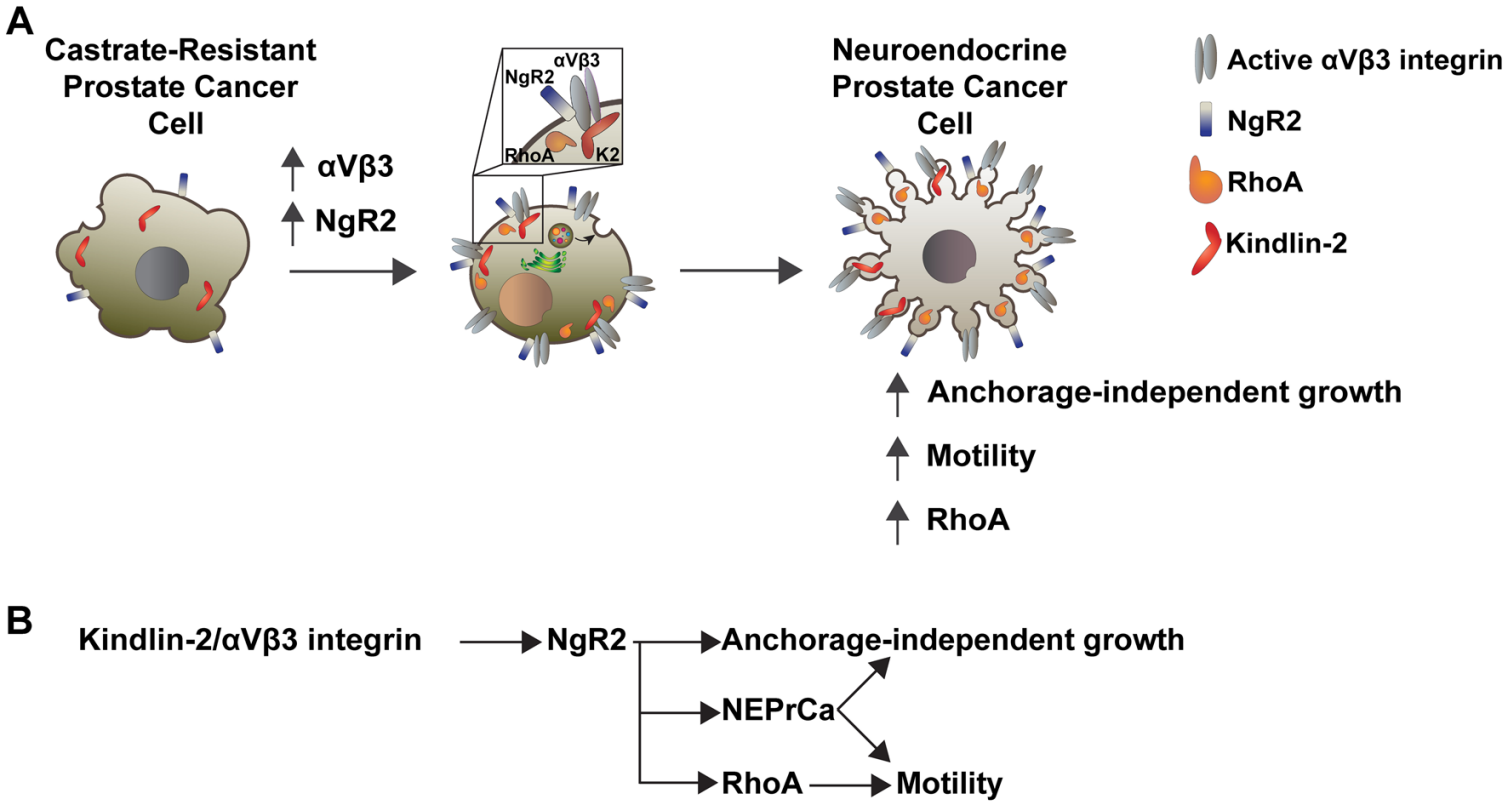
Schematic representation of the pathway described in this paper. (**A**) The αVβ3 integrin, together with its co-activator K2, induces increased levels of NgR2 expression in castrate-resistant prostate cancer cells. In turn, NgR2 regulates the levels of NE markers and stimulates anchorage-independent growth of these cells. NgR2 also upregulates the levels of RhoA that causes increased PrCa cell motility. These cells with increased NgR2 expression are likely to be NEPrCa cells. (**B**) The schematic drawing summarizes our proposed model.

We show that NgR2 regulates NE marker expression in PrCa cells, indicating the involvement of NgR2 in NED. Based on these results showing that αVβ3 integrin and NgR2 drive NED in PrCa and increased motility of NEPrCa and on the fact that the αVβ3 integrin and NgR2 are surface receptors and therefore easily targetable, they are suitable for novel therapeutic approaches in this disease. This approach is in line with a previous study that has shown that the inhibition of the αV integrins by Abituzumab causes the downregulation of several pro-metastatic phenotypes of PrCa cells^62^. However, to prevent the side effects of targeting the αVβ3 integrin, which is expressed in endothelial cells^63,64^, it will be safer to target NgR2, which is downstream of the αVβ3 integrin and of its co-activator, K2. When the expression levels of NgR2 in PC3 cells are reduced using shRNA, we do not detect significant differences in the expression levels of the αVβ3 integrin and K2. On the other hand, exogenous expression of NgR2 does not affect the αVβ3 integrin levels. Overall, these results indicate that NgR2 acts downstream of the αVβ3 integrin and its co-activator K2 and delineate the molecular sequence of this novel pathway, as summarized in Fig. 8.

We also present evidence that RhoA levels are under the control of NgR2; these results are likely to explain the decreased motility of PrCa cells observed upon NgR2 downregulation. This finding is not surprising since members of the NgR family are known to promote RhoA activation^29^ and expression^65^. Furthermore, higher levels of RhoA are found in therapy-resistant PrCa cells^32^, and increased expression and activity of RhoA are essential for invasion and motility of PC3 cells as compared with low invasive PC3 cells^53^. An additional finding is that increased expression of NgR2 and consequently of RhoA requires the αVβ3 integrin to be in an activated state (high affinity/avidity for ligand).

Taken together, our results showing that NgR2 does not affect cell viability, but promotes tumor growth and cell motility, collectively reveal and substantiate a remarkable role for NgR2 in regulating the NE phenotype of PrCa cells. Our findings appear to be highly relevant to NEPrCa given our previous observations that high levels of the αVβ3 integrin^13^ and, as shown here, NgR2 are detected in NEPrCa patients’ tumors as well as in TKO mouse tumors. Furthermore, we expect that NgR2 effect will be highly specific, as compared to the other two members of the NgR family, highlighting the importance of NgR2 in NEPrCa. Finally, it should be stressed that NgR2 is detectable in prostate tumors from the SKO mouse model. Tumors from this model do not express the αVβ3 integrin or the NE marker SYP; therefore, we speculate that both factors, the αVβ3 integrin and NgR2, have to be expressed in order to induce a NE phenotype.

In conclusion, we have identified a novel key mechanism whereby the αVβ3 integrin, and its downstream effector, NgR2, promote a NE and motile phenotype in PrCa (Fig. 8). This study paves new avenues toward reaching a comprehensive mechanistic understanding of integrin-directed signaling during PrCa progression toward a NE phenotype and opens new possibilities for therapeutic strategies and/or risk stratification of PrCa patients.

## Supplementary Information

Supplementary Figures.

Supplementary Information.
