# Supplementary material for: The NOGO receptor NgR2, a novel αVβ3 integrin effector, induces neuroendocrine differentiation in prostate cancer
Source: Sci Rep. 2022 Nov 7;12:18879. doi: 10.1038/s41598-022-21711-5 (PMC9640716; doi:10.1038/s41598-022-21711-5)
Supplement: Supplementary file 1 — Supplementary Figures. [file 41598_2022_21711_MOESM1_ESM.pdf]

The NOGO receptor NgR2, a novel αVβ3 integrin effector, induces neuroendocrine differentiation in prostate cancer

Fabio Quaglia, Shiv Ram Krishn, Khalid Sossey-Alaoui, Priyanka Shailendra Rana, Elzbieta Pluskota, Pyung Hun Park, Christopher D. Shields, Stephen Lin, Peter McCue, Andrew V. Kossenkov, Yanqing Wang, David W. Goodrich, Sheng-Yu Ku, Himisha Beltran, William K. Kelly, Eva Corey, Maja Klose, Christine Bandtlow, Qin Liu, Dario C. Altieri, Edward F. Plow, Lucia R. Languino

Supplementary Material

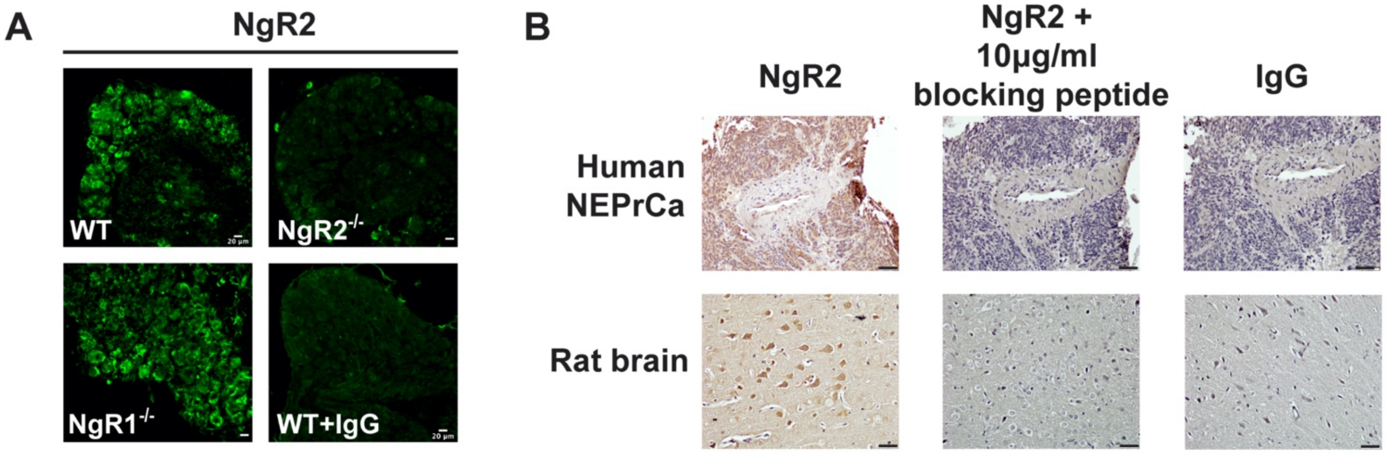

Figure S1. Specificity control of NgR2 immunohistochemical staining. (**A**) Representative immunohistochemical staining of NgR2 of dorsal root ganglion section (n=3) from wild type mice (*top left panel*), NgR2 knockout mice (*top right panel*), and NgR1 knockout mice (*bottom left panel*). IgG on dorsal root ganglion section from wild type mice was used as negative control (*bottom right panel*). (**B**) NgR2 immunohistochemical staining of a needle tumor biopsy from a NEPrCa patient (*top row,* n=1) or rat brain (*bottom row,* n=2) was performed in the presence or absence of a NgR2 Ab (PA5-98577, Invitrogen) incubated with a NgR2 blocking peptide (DSRGRQGGDAPTEDDYWG, 10 μg/ml, Thermo Fisher). IgG was used as negative control. The bars at the bottom right corner of each panel represents 20 μm.

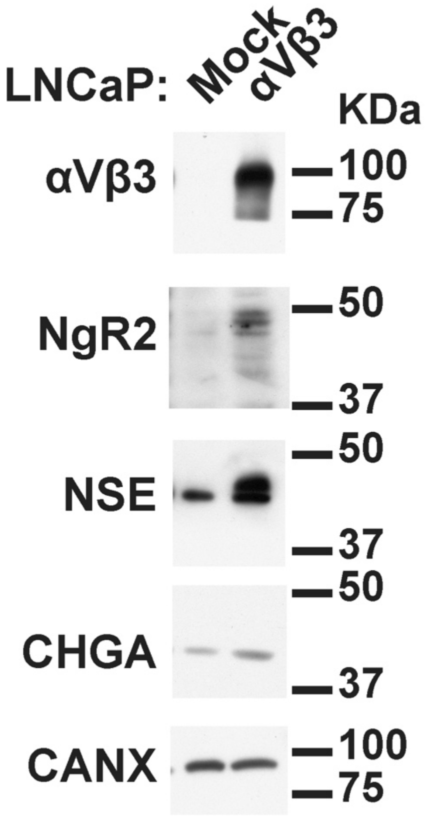

Figure S2. The αVβ3 integrin increases NgR2 expression. Immunoblotting analysis of the expression levels of the αVβ3 integrin, NgR2, and the NE markers chromogranin A (CHGA), and neuron specific enolase (NSE) in LNCaP cells that exogenously express the αVβ3 integrin or their mock control cells (n=2). CANX was used as loading control. The immunoblotting analysis was performed under reducing conditions. NgR2 core protein (45kDa) is predominantly detected in LNCaP.

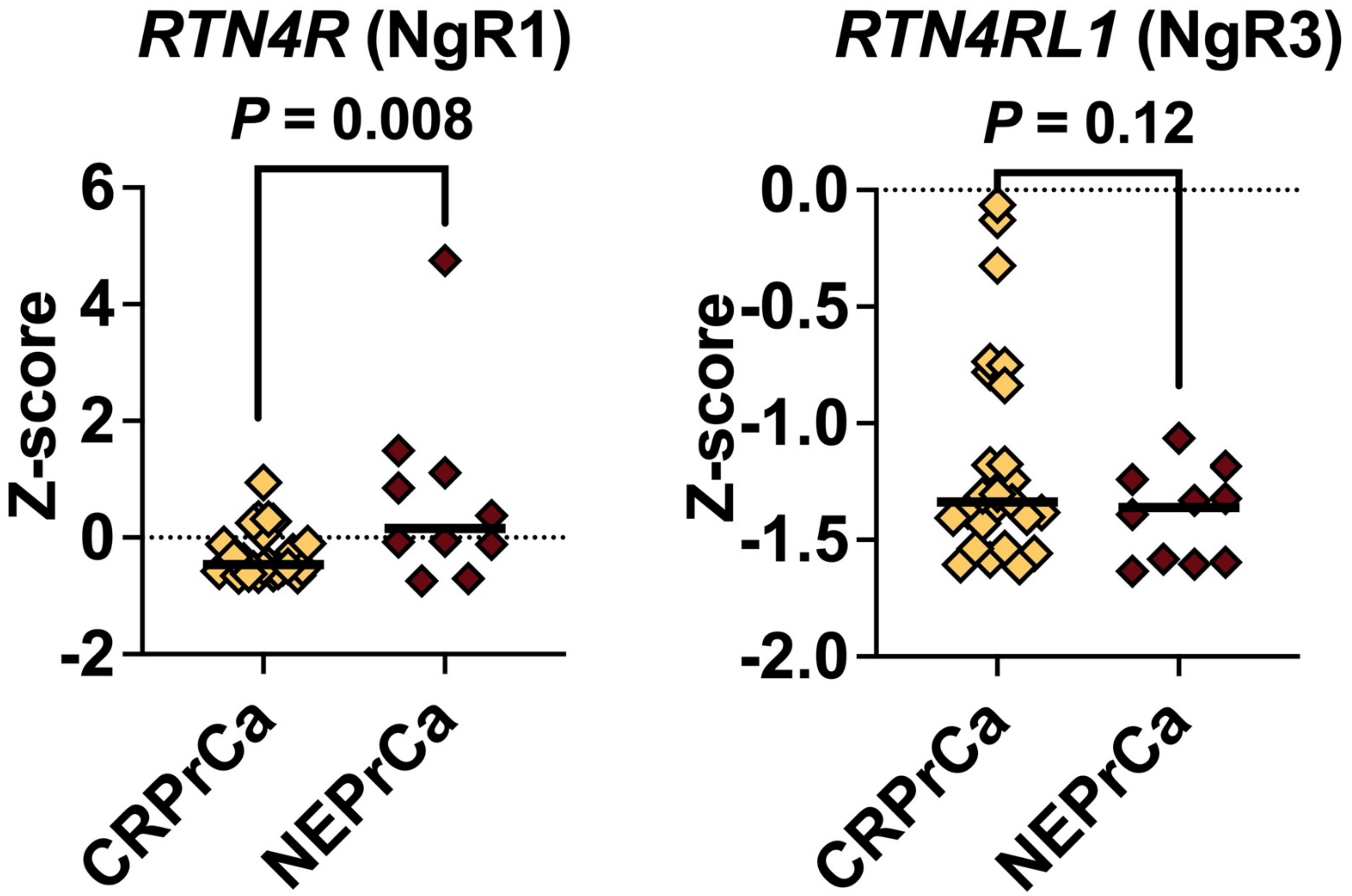

Figure S3. **NgR1 and NgR3 expression in NEPrCa patients.** RNA sequencing analysis of *RTN4R and RTN4RL1* expression for NEPrCa and CRPrCa tumors from cBioPortal (dataset Neuroendocrine Prostate Cancer, Multi-Institute, [20]). Differential expression between the two groups is estimated by student's t-test.

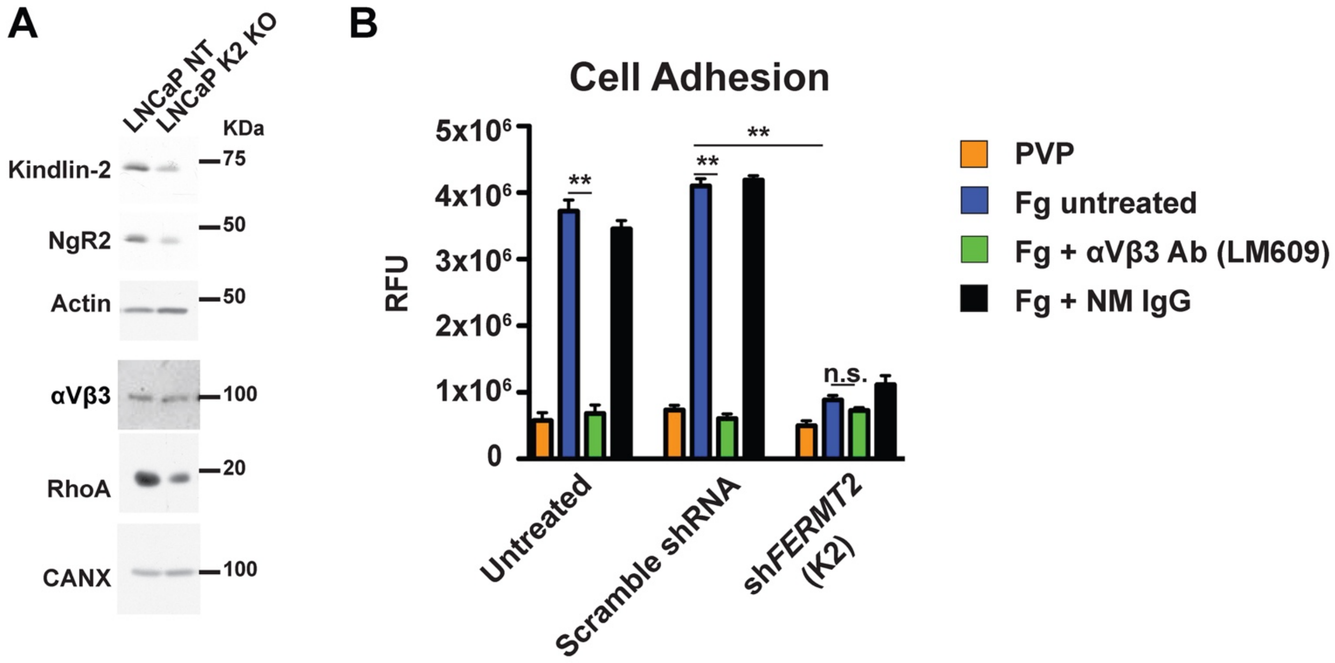

Figure S4. αVβ3-dependent adhesion assay in the presence or absence of *FERMT2* (K2). (**A**) Immunoblotting analysis of the expression levels of αVβ3, NgR2, and Kindlin-2 (*FERMT2*) in the lysates from LNCaP cells in which Kindlin-2 expression was downregulated using sgRNA targeting FERMT2 (the gene responsible for Kindlin-2) (n=1). Actin or CANX was used as loading control. The immunoblotting analysis was performed under reducing conditions. (**B**) Adhesion of PC3 cells to control PVP and Fg in the absence or presence of either αVβ3 blocking Ab (LM609) or control non-immune mouse IgG. Values are reported as Relative Fluorescence Units (RFU); *P*-values were calculated by unpaired t-test using GraphPad Prism. NT, Non-Targeting; n.s., non-significant; ** $P < 0.005$.
