# Supplementary material for: The NOGO receptor NgR2, a novel αVβ3 integrin effector, induces neuroendocrine differentiation in prostate cancer
Source: Sci Rep. 2022 Nov 7;12:18879. doi: 10.1038/s41598-022-21711-5 (PMC9640716; doi:10.1038/s41598-022-21711-5)
Supplement: Supplementary file 2 — Supplementary Information. [file 41598_2022_21711_MOESM2_ESM.pdf]

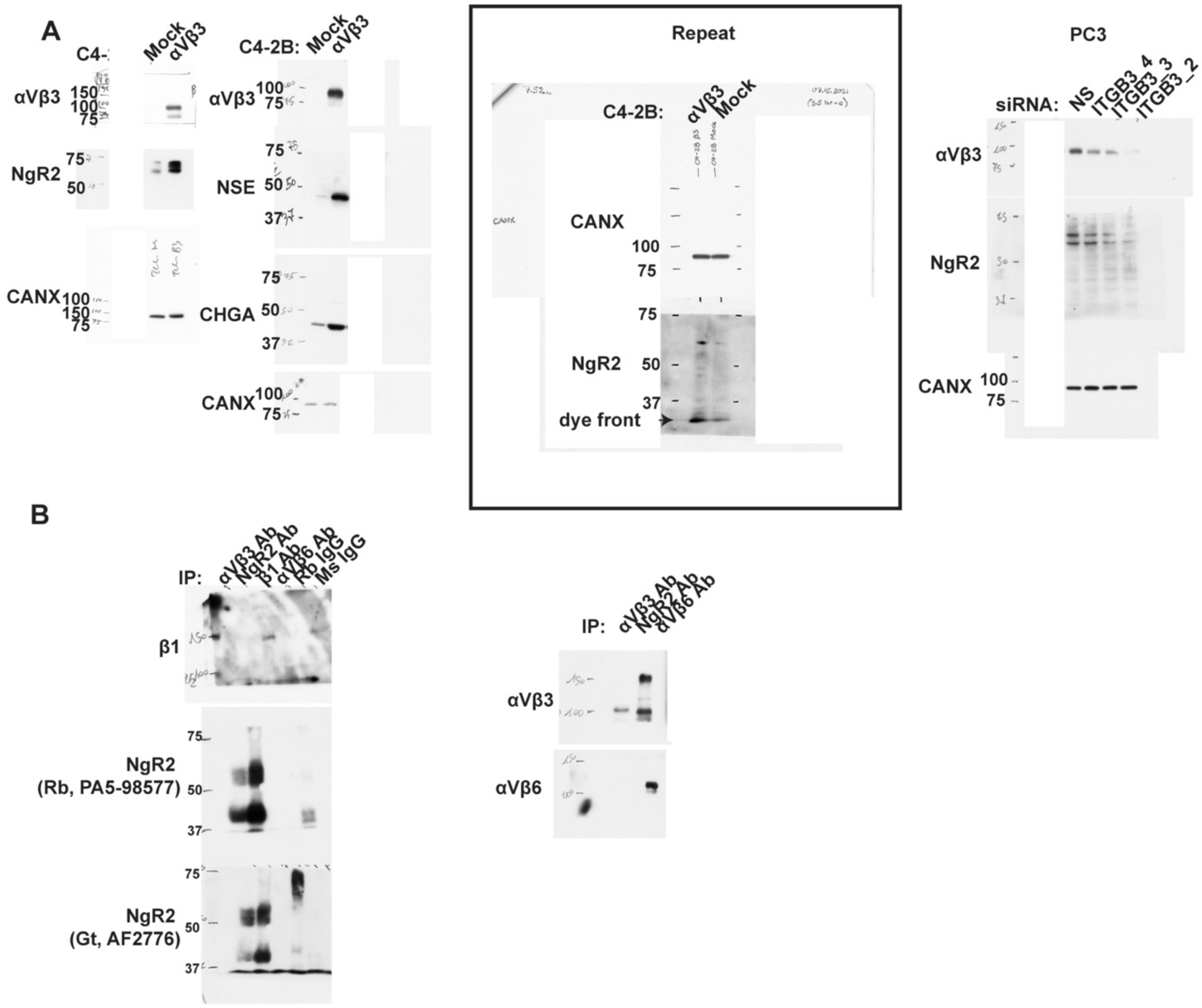

Originals of Fig. 1

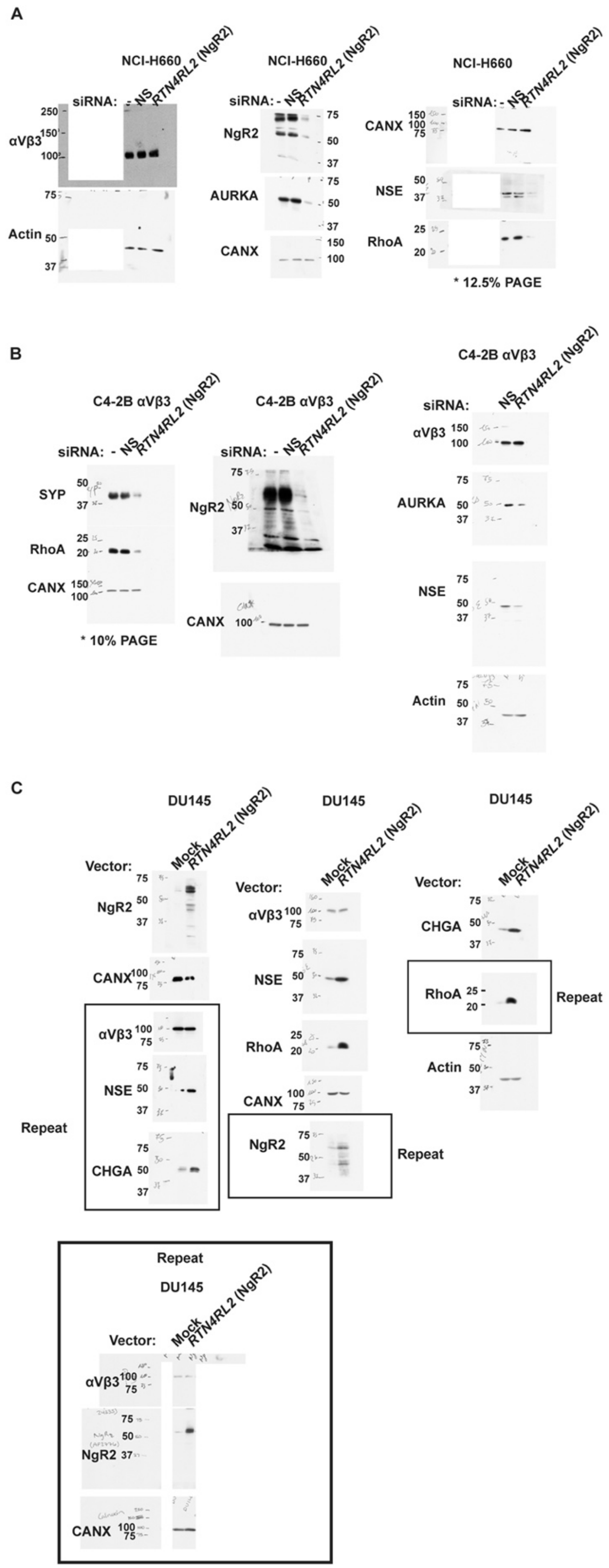

Originals of Fig. 4

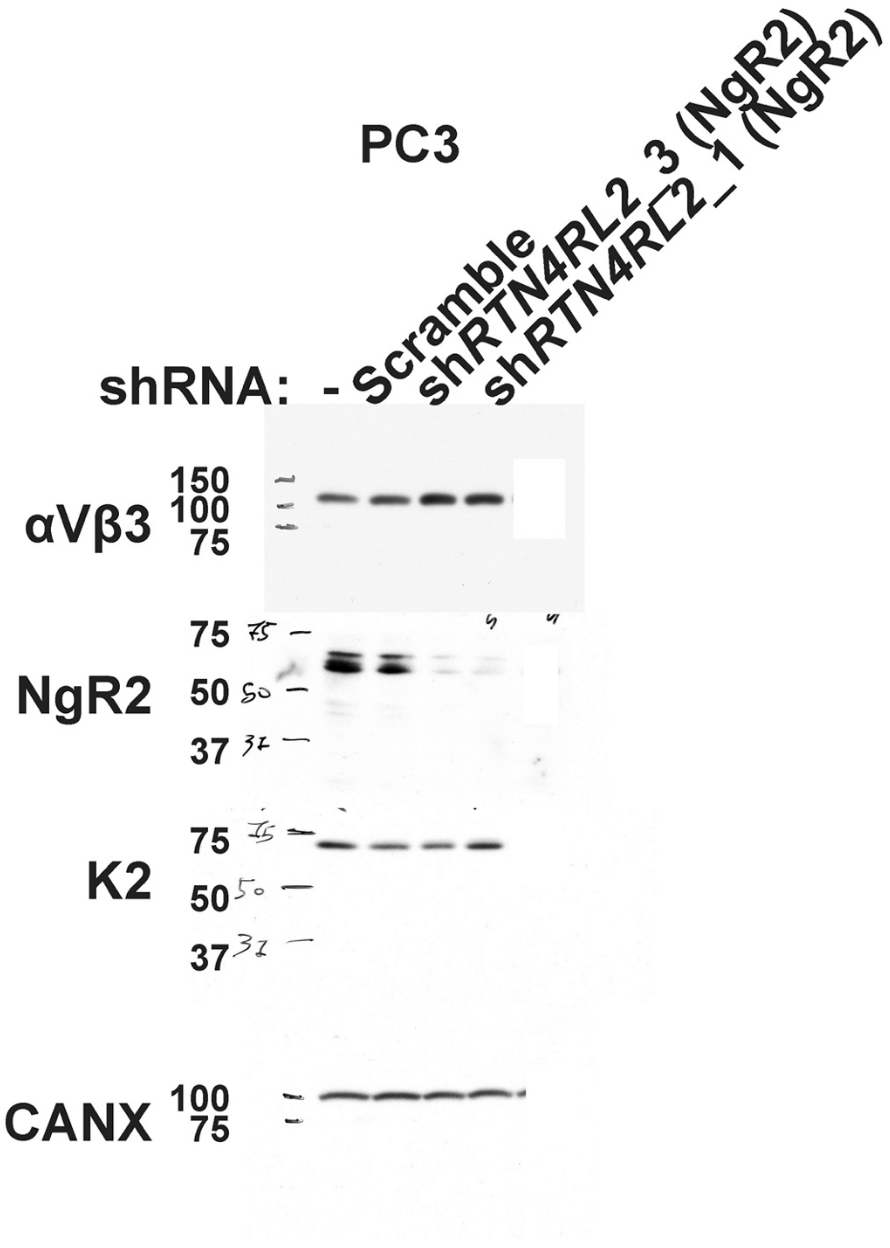

Originals of Fig. 5

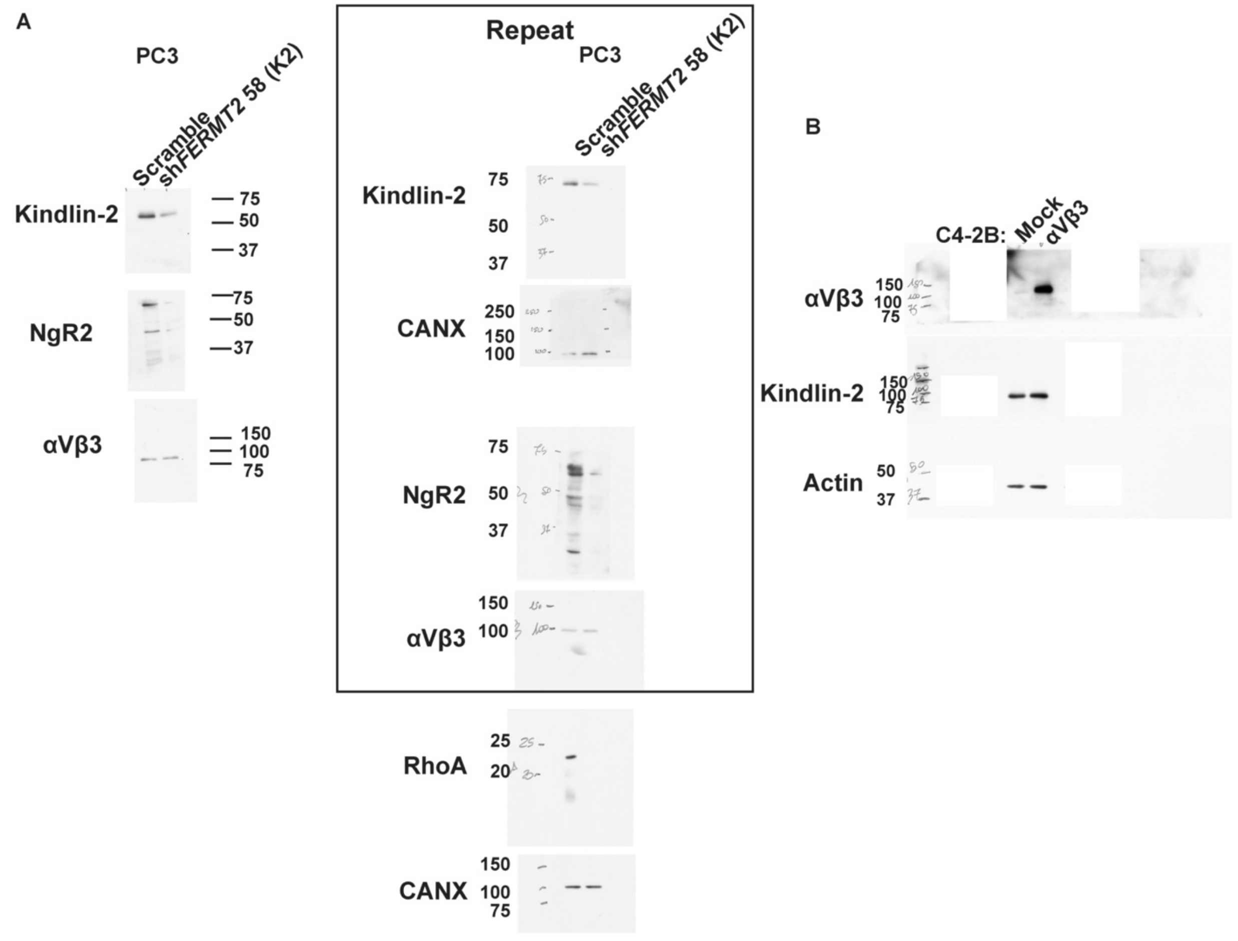

Originals of Fig. 7

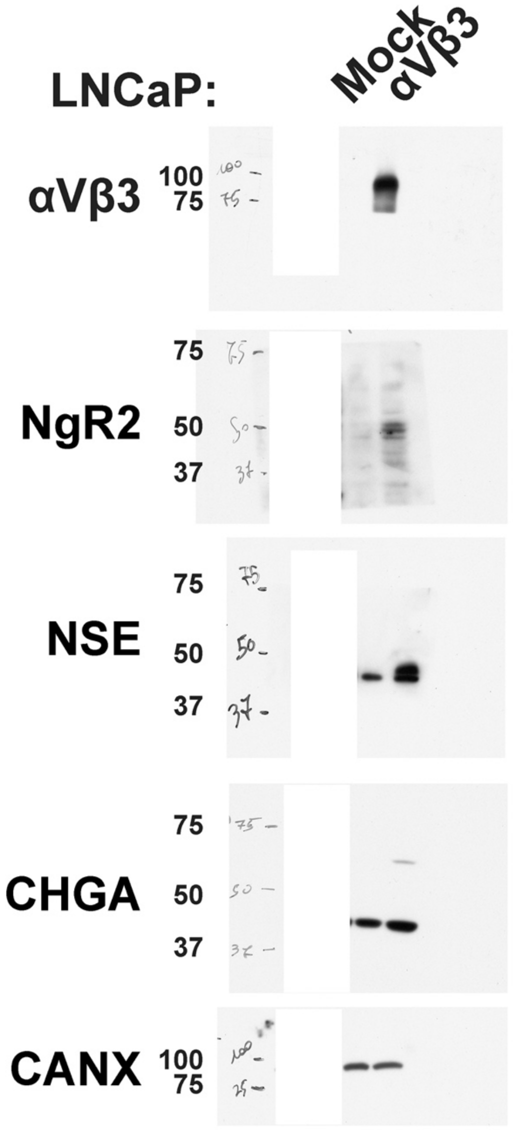

Originals of Fig. S2

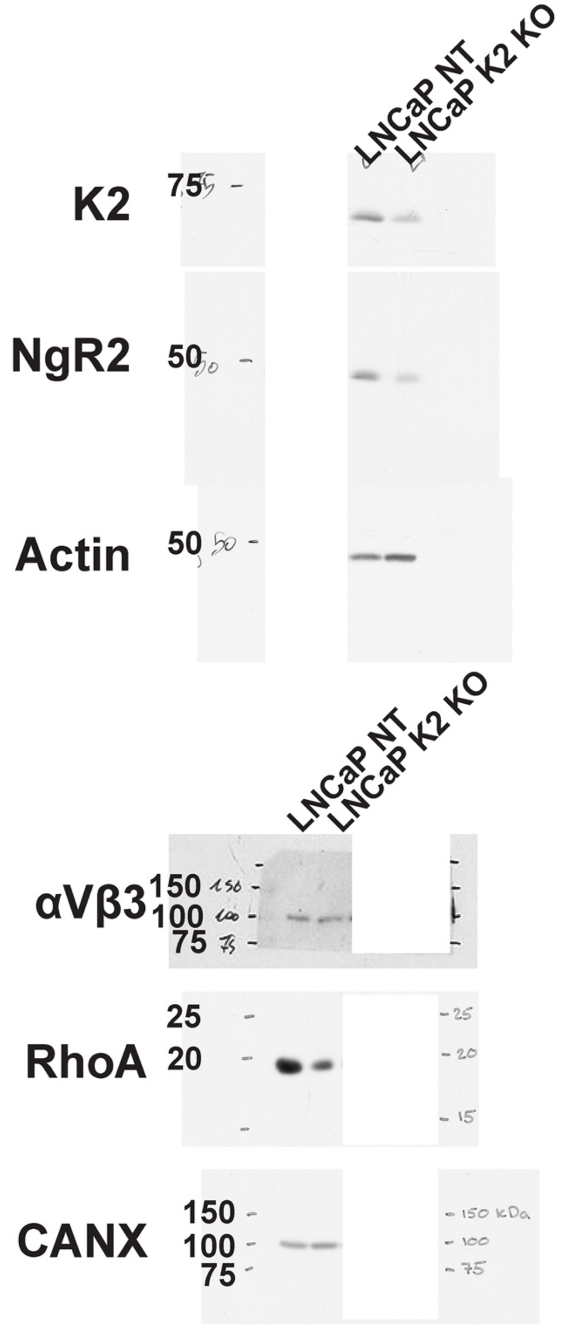

Originals of Fig. S4
